# Supplementary figures and images for: Quantitative Phosphoproteomics of cipk3/9/23/26 Mutant and Wild Type in Arabidopsis thaliana
Source: Genes (Basel). 2021 Nov 4;12(11):1759. doi: 10.3390/genes12111759 (PMC8623713; doi:10.3390/genes12111759)

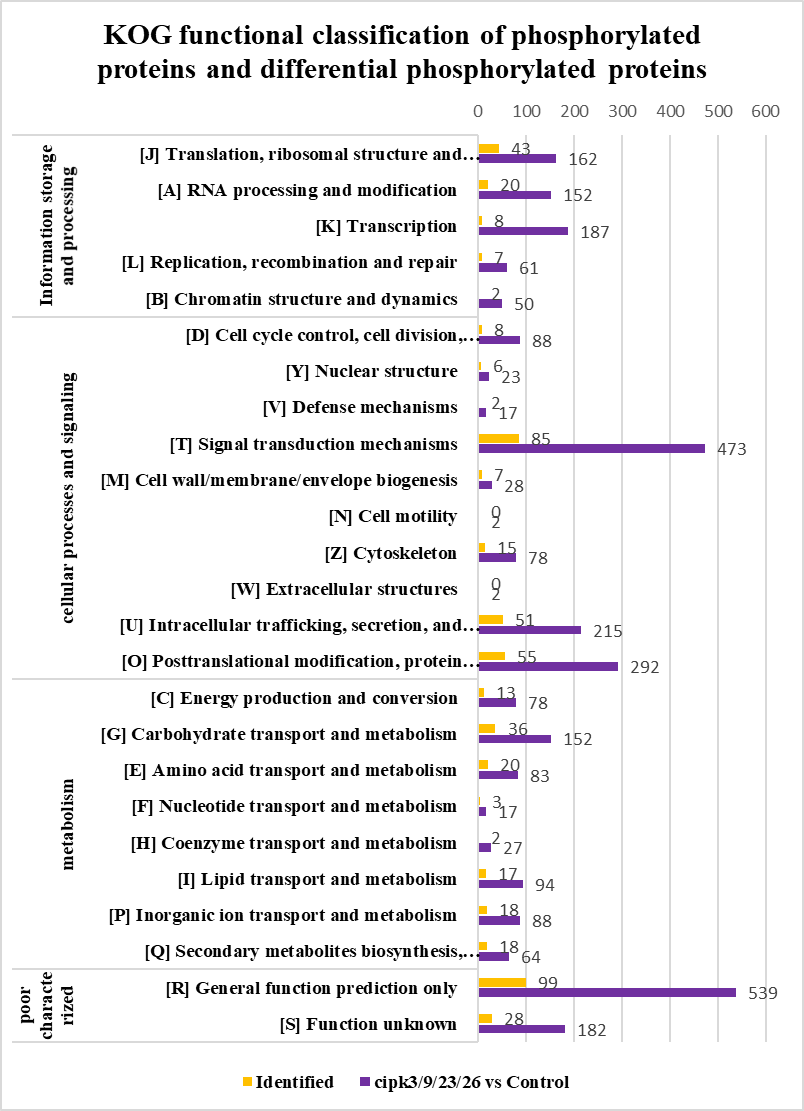

Supplement: Supplementary file 1 [file genes-12-01759-s001.zip › Supplementary material/Supplementary Figure S1.png]
